# Empirical study using network of semantically related associations in bridging the knowledge gap

**DOI:** 10.1186/s12967-014-0324-9

**Published:** 2014-11-27

**Authors:** Vida Abedi, Mohammed Yeasin, Ramin Zand

**Affiliations:** The Center for Modeling Immunity to Entering Pathogens, Nutritional Immunology and Molecular Medicine Laboratory, Virginia Bioinformatics Institute, Virginia Polytechnic Institute and State University, Blacksburg, VA 24060 USA; Department of Electrical and Computer Engineering, Memphis University, Memphis, TN 38152 USA; College of Arts and Sciences, Bioinformatics Program, Memphis University, Memphis, TN 38152 USA; Department of Neurology, University of Tennessee Health Science Center, Memphis, TN 38163 USA

**Keywords:** Knowledge discovery, Hypothesis generation, Literature mining, Ontology mapping, PubMed, Medical subject headings (MeSH), Multi-gram dictionary, Latent semantic analysis (LSA), Network of association, Semantic associations

## Abstract

**Background:**

The data overload has created a new set of challenges in finding meaningful and relevant information with minimal cognitive effort. However designing robust and scalable knowledge discovery systems remains a challenge. Recent innovations in the (biological) literature mining tools have opened new avenues to understand the confluence of various diseases, genes, risk factors as well as biological processes in bridging the gaps between the massive amounts of scientific data and harvesting useful knowledge.

**Methods:**

In this paper, we highlight some of the findings using a text analytics tool, called ARIANA - Adaptive Robust and Integrative Analysis for finding Novel Associations.

**Results:**

Empirical study using ARIANA reveals knowledge discovery instances that illustrate the efficacy of such tool. For example, ARIANA can capture the connection between the drug hexamethonium and pulmonary inflammation and fibrosis that caused the tragic death of a healthy volunteer in a 2001 John Hopkins asthma study, even though the abstract of the study was not part of the semantic model.

**Conclusion:**

An integrated system, such as ARIANA, could assist the human expert in exploratory literature search by bringing forward hidden associations, promoting data reuse and knowledge discovery as well as stimulating interdisciplinary projects by connecting information across the disciplines.

## Background

**Figure 1:**
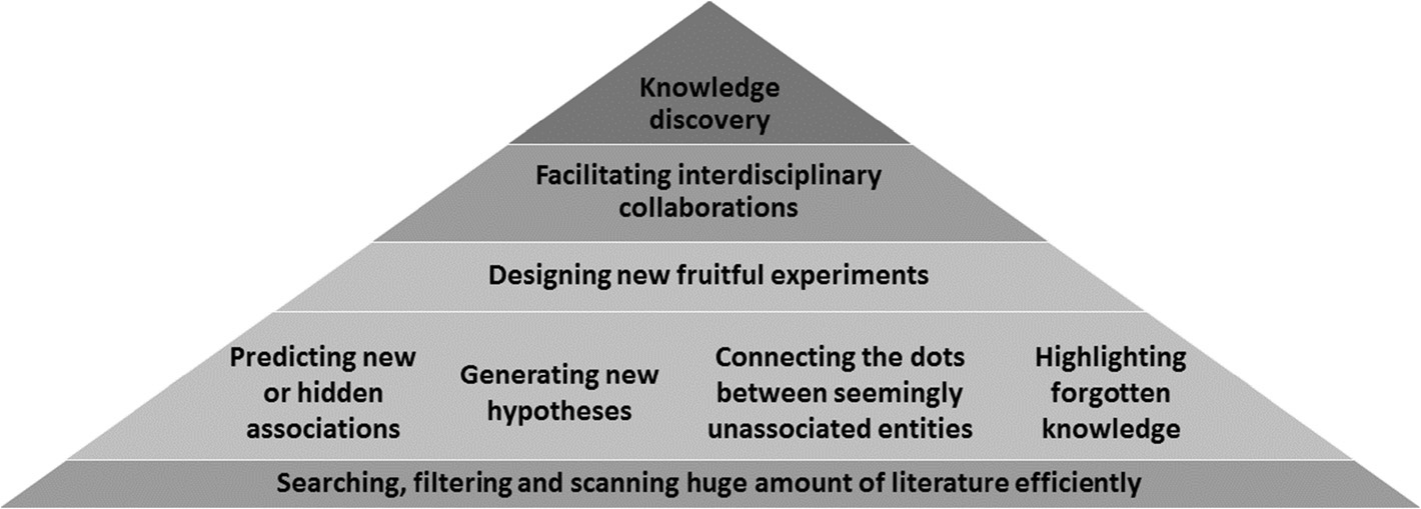
**Array of applications of ARIANA.**

Strategic reading, searching, and filtering have been the norm in gaining perspective from the “ocean of data” in the field of biomedicine. Intriguingly, the information overload has contributed in widening the knowledge gap. On an average day in 2013, approximately 3 million searches were performed on the PubMed web site, and an additional 3 million searches were done by scripts (e.g., by application programming interfaces or APIs) [1]*.* It is widely acknowledged that efficient mining of biological literature could provide a variety of services [2] such as assisted curation of literature [3], hypothesis generation [4], association prediction [5] or semantic sensitive knowledge discovery (Figure 1). Traditionally, literature mining tools focus on text summarization and clustering techniques [6] with the goal of reducing the data overload and ability to read and synthesize more information in a shorter time. In recent years effective LDA [4] as well as LSA [7]-based techniques have also been used with great success to formulate new hypotheses and generate connections from existing literature. It was argued that a text analytic tool capable of extracting network of semantically related associations may help in bridging knowledge gap by using human’s unique visual capacity and information seeking behavior. For instance in a study, 16,169 articles were chosen to create a visual representation of main concepts, creating a visual maps of verbal information [8]. In that analysis, it was found that “*verbal presentation offers more precise information […], whereas the visual presentation offers a more flexible style of exploration that better shows multiple, fuzzy, and intermixed and complexly patterned relations among the documents* [8]”. In addition, literature mining tools that can capture the semantic relationship could in principle connect disjoints entities between different research fields.

**Figure 2:**
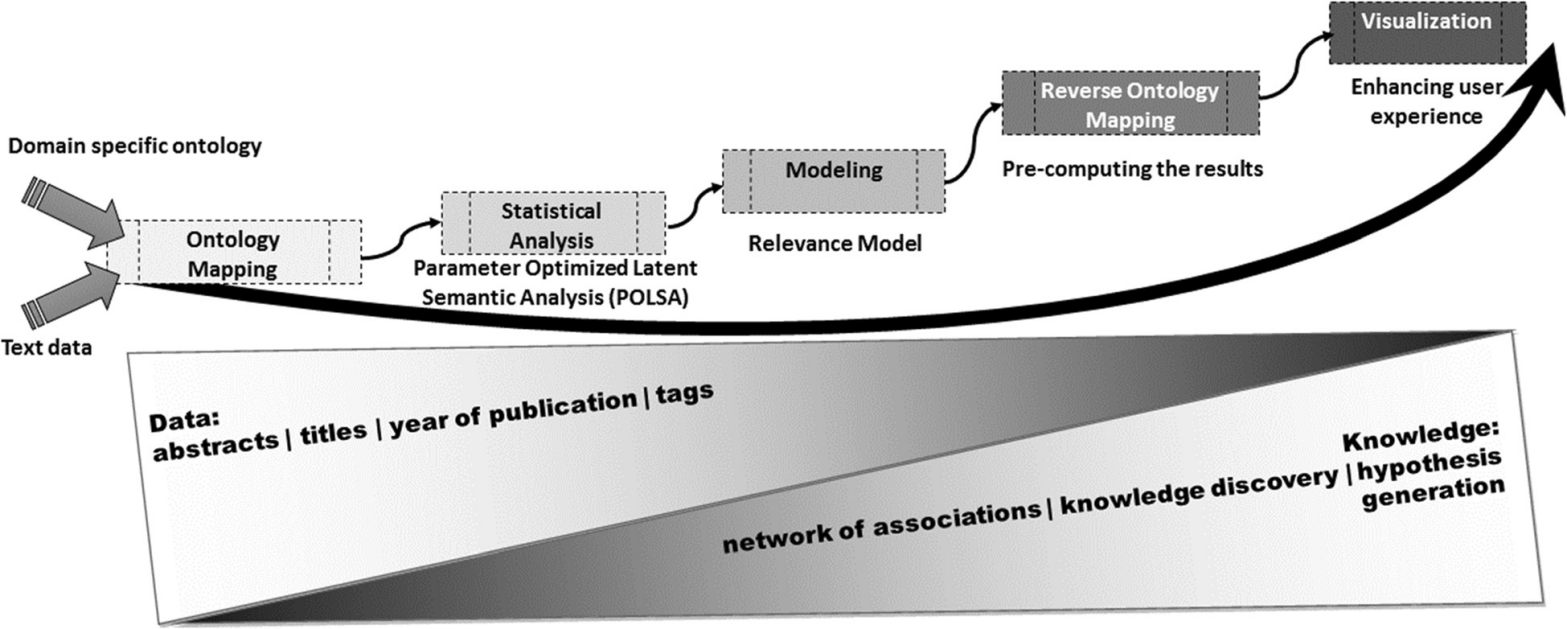
**Road-map to find the network of semantic associations in bridging the gap between the production and consumption of bio-medical data.**

A well designed literature mining tool can be used to further the understanding of potential confluence between various diseases, drugs, genes, proteins and other risk factors. To be effective in bridging the monotonically increasing gaps between the data production and its utilization, such a tool should efficiently map domain specific information and capture network of semantic-sensitive association and provide effective and seamless visualization (Figure 2). This paper highlights the key findings of empirical studies performed using a knowledge discovery tool, called ARIANA.

## Methods

ARIANA [9] is a software system that is designed to capture *“crisp semantic associations”* among bio-medical concepts of interest and provide scalable Web-Services (Figure 2). It integrates semantic-sensitive analysis of text data through ontology mapping with database search and advanced visualization of the network of semantically related associations that can be easy to collapse and expand, allowing the user to have a global view of the results or to focus on a sub-network. As an integrative tool, goals of ARIANA are to find the network of semantic associations in bridging the gap between the production and utilization of data, disambiguate the domain specific entities, provide robust results to a broad range of queries and, deliver a scalable Web-Service using state-of-the-art technology.

**Figure 3:**
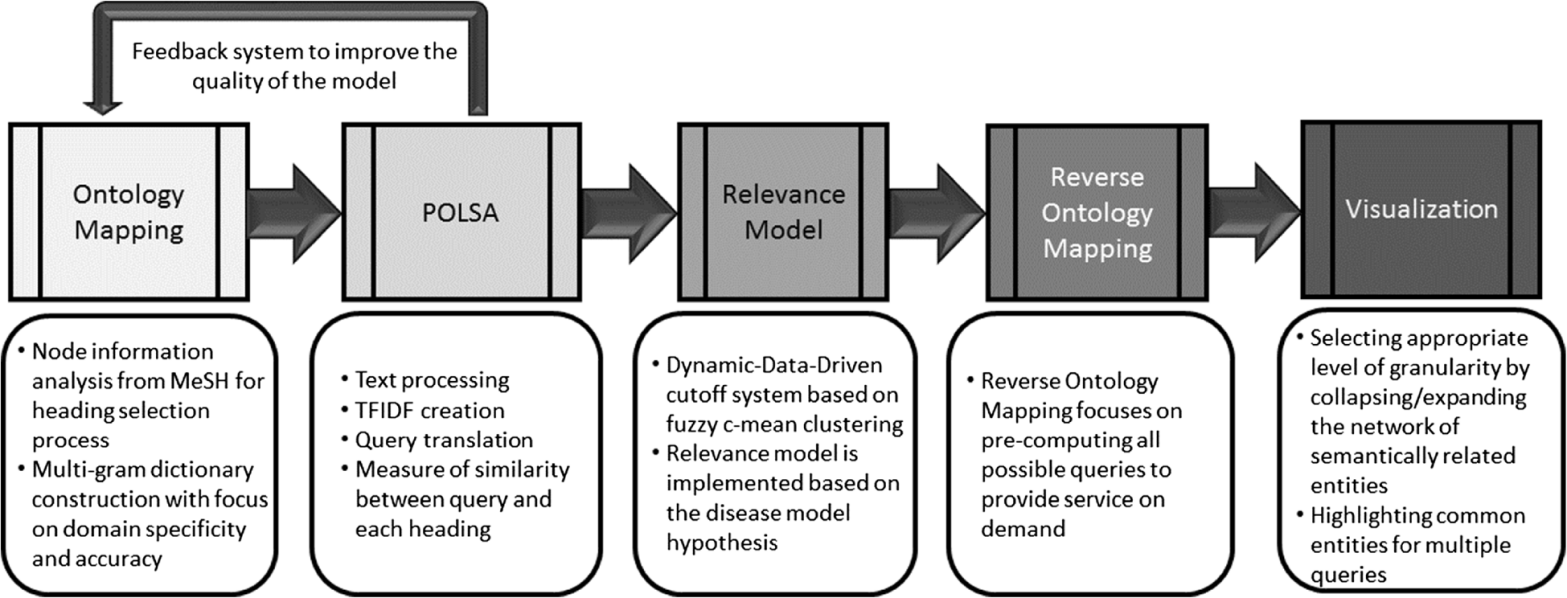
**Data processing stages of ARIANA.**

**Figure 4:**
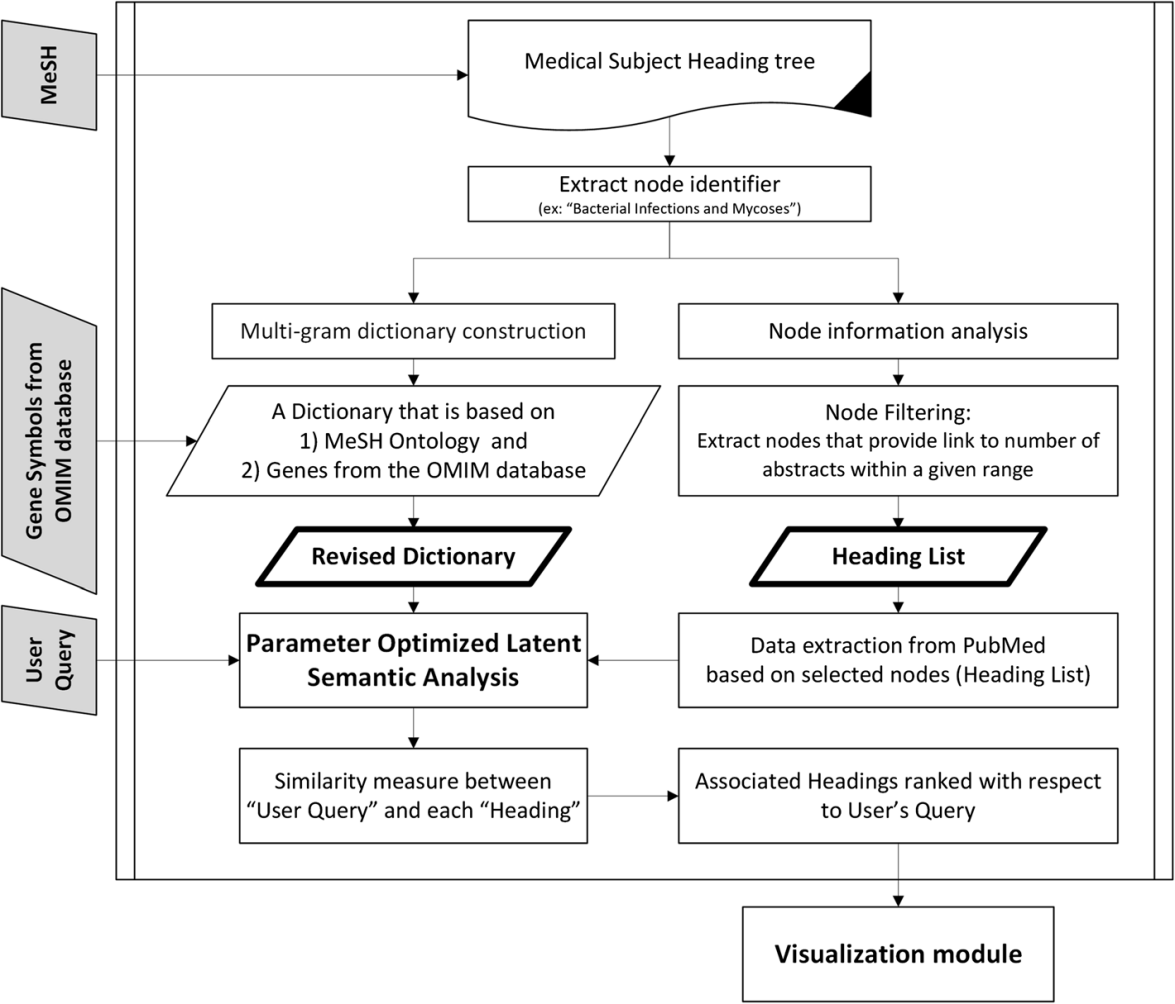
**ARIANA’s core architecture.**

Evaluating knowledge discovery tools remain challenging due to inherent subjective nature of the findings. However, empirical studies can be used to illustrate the efficacy of such tools. The semantic model of ARIANA (Figure 3) was significantly expanded to include 8,700,000 representative abstracts from the PubMed [1] database covering fifty years of literature, and 2,545 hierarchically-structured Headings^b^ from MeSH (Medical Subject Headings) ontology [10]. The Heading selection process is implemented based on heuristics to capture a representative and balanced data. The main constraint being that half of the selected headings for the model have to be from the Disease category since the main focus of the study is to identify disease related risk factors. The main feature used to select the headings was the number of abstract in each category. For instance, categories with fewer than 1,000 or greater than 50,000 abstracts were not considered, as they represent very specific or very general topics. For instance, there are 1,828 headings from the Disease category (category C), 475 headings from Chemical and Drugs category (category D), and the remaining headings from categories F, G, I, J, M and N [10]. Performing Ontology Mapping (OM) on the 2012 version of the MeSH and combing to that gene symbols, from the OMIM (Online Mendelian Inheritance in Man) [11] database generated a context-specific and multi-gram dictionary with 17,074 terms (for instance, the term “yellow virus fever” is a medical multi-gram term with very specific meaning extracted from parsing the MeSH)^c^. The latter is critical for efficient mapping of domain knowledge to the semantic space with layer of genetic information. ARIANA adopts the POLSA [12] to capture direct as well as indirect statistical associations among the dictionary terms. In the POLSA model, term-frequency inverse-document-frequency (TF-IDF) matrix was used to generate the *encoding* matrix and the dimensionality was reduced to cover 95% of the total energy (dimensionality was reduced from 2,545 to 1,400 headings to create the encoding matrix). Furthermore, the multi-gram dictionary also captures higher order associations among the different entities. A Relevance Model (RM), implemented using fuzzy c-mean clustering, is introduced to logically group the ranked results of the user’s query to generate hypothesis [5] and facilitate the identification of highly related entities in a Dynamic-Data-Driven (DDD) fashion [5]. Fuzzy c-means clustering is applied to group the ranked headings into three groups (highly associated, possibly associated or unknown) [9]. The cut-off values estimated through this process are DDD and subject to change as the dataset expands [9]. Reverse Ontology Mapping (ROM) was introduced to generate networks from the semantically related entities that are considered relevant. The ROM was used to map the terms back to the MeSH and create a network of associations. All possible queries were pre-computed, stored in a hash table and, linked with the visualization module in order to provide services on demand. Finally, to present the results in a graphical representation, JavaScript Object Notation (JSON*)* objects are created, and the D3 library^d^ is used to implement the collapsibility and expandability as well as flexibility to stretch the objects to provide better visual clarity for each node. Figure 4 highlights the main steps in the data extraction and analysis of the system.

## Results and discussion

Empirical study using the improved ARIANA was performed to identify network of associations with single as well as multiple query words. Representative of the findings are succinctly summarized below to illustrate the utility of such system in discovering unknown interactions and also to generate robust hypothesis by connecting the information from interdisciplinary fields. However, in order to extract hidden knowledge for a single vital query, such as the case for the asthma study at John Hopkins, it is imperative to not only focus on the graph representation but also extract the raw association scores and investigate entities with weaker level of associations. In essence, with no direct evidence in the literature, weaker yet positive associations tend to provide key indication for further in-depth investigation.

Case Study on (lethal) drug interactions in designing experiments: In 2001, an asthma research team at the John Hopkins University used the drug hexamethonium on a young healthy volunteer that ended in a tragic death due to pulmonary inflammation and fibrosis. Office for Human Research Protections of the US Department of Health and Human Services faulted the investigators for ignoring published information regarding the lung toxicity of the drug. In an internal investigation [13], the committee noted “The principal investigator subsequently stated to the investigation committee that he had performed a standard PubMed search”. The committee panel referred to a number of studies, in addition to one case-report published in 1955 [14], that have reported an association between hexamethonium and pulmonary fibrosis. In that case report [14], a 28 year old woman died after receiving hexamethonium over a period of six months. Even after these two tragedies, the association between, hexamethonium and pulmonary fibrosis, or fibroma are still not evident with a keyword search from PubMed. The second tragedy was never published as a case report; nonetheless, the autopsy report as well as news broadcasts are available on the internet. This tragedy gained media’s attention because it could have been prevented. In our test, ARIANA provides evidence for such associations. This knowledge was extracted even though the constructed core database contains publications from 1960 to 2012. Out of 2,545 concepts selected from the MeSH, “*Scleroderma, Systemic”*, “*Neoplasms, Fibrous Tissue*”, “*Pneumonia*”, “*Fibroma*”, and “*Pulmonary Fibrosis*” were ranked as the 13^th^, 16^th^, 38^th^, 174^th^ and 257^th^ ranked-concept, respectively. If the researchers had access to such knowledge discovery tool, capable of identifying novel associations, this investigator would likely have performed additional in-depth research before using this drug on a healthy subject. A network view of the query hexamethonium indicates that the top seven associations are relevant; however, due to the nature of the investigation, we expect the weaker associations to provide key information worth further in-depth verification by experts.

Identification of network of semantically related entities with a single or double query can uncover hidden knowledge and facilitate data reuse among other things. Alzheimer’s disease (AD) is a debilitating disease of the nervous system, mostly affecting the older population. ARIANA captured some of the obvious associations such as Tauopathies; Proteostasis Deficiencies; Amyloidosis; Cerebral Arterial Diseases; Multiple System Atrophy; Agnosia. It also identified some of the less obvious associations such as Tissue Inhibitor of Metalloproteinases [15,16]. Using Tuberculosis (TB) as a second query, a common entity was recognized to be linked to both AD and TB. “Proteostasis Deficiencies > Amyloidosis” is highly related (cosine score of 0.5651) to TB and moderately related (cosine score of 0.0734) to AD. Further investigation by expert revealed that AD and TB could be indirectly related through MMP (Matrix metalloproteinases) gene family members. MMPs are zinc-binding endopeptidases that degrade various components of the extracellular matrix [17,18]. MMPs are believed to be implicated in TB by the concept of a matrix degrading phenotype [19]. Various studies in human cells, animal models as well as gene profiling studies support the association of MMPs and TB and involvement of TB-driven lung matrix deconstruction [20-24]. MMPs are also implicated in AD [25] but in a more positive way. In fact MMP proteins can breakdown the amyloid proteins [26] that are present in the brain of the AD patients. There is literature evidence for the link between MMP genes and AD, and similarly between MMP genes and TB; however, the connection between AD and TB through the MMP genes is extracted by a global analysis of the literature.

Finally, ARIANA can be used by expert to perform global literature search using 17,074 different queries, and these include diseases, risk factors, biomedical entities and biological processes. Two additional search results from the system are summarized: 1) *Query term*: **CD4**. The five top associated headings are i) cyclin D, ii) retinal pigments > opsins, iii) human immunodeficiency virus, iv) beta-endorphin, and v) alloys > steel. 2) *Query term*: **Helicobacter pylori**. Among the top associated headings are i) apolipoproteins B, ii) adrenergic alpha-agonists, iii) isonicotinic acids, iv) oral fistula, v) identification (psychology) > gender identity and vi) diabetes mellitus, type 1. All these associated MeSH terms with the two queries have supporting evidence in the literature, even if at first some might seem unrelated. Exploring such associations, and even those that are at slightly weaker levels could provide valuable opportunities in knowledge discovery and hypothesis generation.

ARIANA is a LSA-based technique that integrates ontology mapping and advanced visualization technique to provide a global view of the knowledge that is buried in the ocean of literature. ARIANA has many advantages, such as scalability, context specificity, robustness and language-independence; however, the system has also some limitation. For instance, it is well agreed that an LSA-based technique is computationally intensive because of its utilization of Singular Value Decomposition step [7]. However, with higher computing power and the possibility to perform parallel computing this limitation can soon be overcome. A second limitation of this method is in its use of bag-of-word model, where ordering of words is lost; ARIANA uses multi-gram dictionary which alleviates this problem to some extent while still proving scalability. Finally a major different between LSA based techniques and part-of-speech tagging is LSA’s inability to provide direct link to the specific publication that was the source of the identified association. We are currently working to address this specific limitation which can also be very valuable to the broader field of computational science.

## Conclusion

An array of text-analytics tools [2,4,6,7] are being developed to answer and solve specific problems when dealing with biomedical literature that is increasing at an unprecedented rate. There are three main features that distinguish this work from closely related work such as Bio-LDA [4]: 1) modularity in terms of concept selection (from MeSH), 2) multi-gram dictionary construction (providing context specificity and enhanced semantics) and 3) scalability (where 50 years of literature from PubMed is analyzed). However, the system has its own limitations as stated in the discussion; our group along with others in the computational field [27-29] are actively working towards addressing these limitations..

Finally, network of semantically related associations is critical to understand the confluence between diseases, drugs, genes and risk factors. To be effective, such a tool must be efficient, robust, scalable, and useable in finding meaningful information beyond literature mining. It is the features like disambiguation of domain specific entities, flexibility in terms of visualization, broadness in coverage, robustness in modeling and scalability in providing array of Web services that made ARIANA an important tool to bridge the gap between data and knowledge.

### Availability

Software is available with properly executed end users licensing agreement (EULA) at http://www.ARIANAmed.org^a^.

## Endnotes

^a^Requests for an account should be made to VA (abedi@vbi.vt.edu) or to MY (myeasin@memphis.edu).

^b^The list of 2,545 hierarchically-structured Headings used in the model is available upon request.

^c^The multi-gram dictionary used in the study is available upon request.

^d^http://d3js.org/.

## Abbreviations

ARIANA: Adaptive robust and integrative analysis for finding novel associations
AD: Alzheimer’s disease
DDD: Dynamic-data-driven
LSA: Latent semantic analysis
MeSH: Medical subject headings
MMP: Matrix metalloproteinases
OM: Ontology mapping
OMIM: Online mendelian inheritance in man
POLSA: Parameter optimized latent semantic analysis
RM: Relevance model
ROM: Reverse ontology mapping
TB: Tuberculosis
TF-IDF: Term-frequency inverse-document-frequency
